# The outcome of expandable titanium mesh implants for the treatment of multi-level vertebral compression fractures caused by multiple myeloma

**DOI:** 10.1051/sicotj/2021026

**Published:** 2021-04-30

**Authors:** Surya Gandham, Abdurrahman Islim, Saud Alhamad, Sathya Thambiraj

**Affiliations:** 1 Department of Spinal Surgery, The Royal Liverpool University Hospital Trust Prescot Street L7 8XP Liverpool United Kingdom

**Keywords:** Vertebral compression fractures, Myeloma, Expandable titanium mesh implants, Osseofix^®^

## Abstract

*Background*: Painful vertebral compression fractures (VCFs) in myeloma patients severely reduce quality of life. Currently, the International Myeloma Working Group (IMWG) and National Institute of Clinical Excellence NICE advocate the use of either balloon kyphoplasty or vertebroplasty in the management of these fractures. *Methods*: All patients with VCFs and myeloma who adhered to the IMWG indications for vertebral augmentation were treated with the Osseofix^®^ implant. Visual analogue scores (VAS) and Oswestry disability index (ODI) were taken preoperatively and at least one year following surgery. Cobb angle and implant migration were measured on lateral standing radiographs. *Results*: Sixteen patients (average age 62, SD = 11.6) consisting of 82 levels (range 3–8) were stabilised with no perioperative complications or revisions at one year. There was an improvement in patient-reported outcomes with the median preoperative VAS of 8.6 (IQR 7.3–10.0) reducing to 3 (IQR 1.0–4.0) after one year (*P* < 0.001) whilst an average improvement of 31.4 (SD = 19.6) points in the ODI scores was reported (*P* < 0.001). There was no significant collapse or implant failure at one year with a greater improvement in the VAS/ODI score, when more implants were used *(P* = 0.049 and 0.008, respectively*).* The average length of stay was 2.2 days (SD = 1.7). *Conclusion*: The use of the Osseofix^®^ implant in VCFs caused by multiple myeloma has shown a statistically significant improvement in both pain and outcome scores. There were no complications or significant radiological deterioration of spinal alignment over the course of a year.

## Background

Multiple myeloma (MM) is a clonal B-cell disorder characterised by proliferation and accumulation of B-lymphocytes and plasma cells in the bone marrow and has an incidence of 6/100,000 in western populations [1]. MM has a predilection for the spine due to the high hematopoietic marrow content of the vertebrae and offers an ideal site for local infiltration and growth of neoplastic plasma cells [2]. Through various signalling pathways, osteoclasts are preferentially activated and the homeostatic balance of bone remodelling shifts towards resorption [3]. This leads to localised osteoporosis and may lead to vertebral compression fractures (VCFs). VCFs are also compounded by the use of high-dose steroids in the treatment of MM [4].

Multiple VCFs not only cause pain for the patient but can result in sagittal and coronal plane deformities which can adversely affect functional status, lung function and increase pulmonary complications [5]. If non operative management fails, there are various vertebral augmentation techniques that can be used [6]. First-generation vertebral augmentation consists of injecting low-viscosity polymethyl methacrylate (PMMA) bone cement at high pressure directly into the collapsed vertebral body and has shown to restore its strength and prevent further kyphosis [7]. The most common complications associated with this technique are PMMA leakage into surrounding tissues – 20%; paravertebral vein embolism – 13%; intradiscal leakage – 8% and PMMA leakage into the spinal canal – 0.8% [8].

These complications prompted the creation of second-generation techniques such as balloon kyphoplasty. This procedure involves the inflation of a balloon catheter inside the collapsed vertebral body which restores its height before the fracture is stabilised with bone cement. The balloon creates a cavity inside the vertebral body into which a more viscous cement can be injected at lower pressure, thus considerably reducing the risk of leakage [9]. Despite improving cement leakage, both treatment strategies allow incomplete fracture reduction or loss of the restored height in the time frame between reduction with balloon deflation and cement application [10].

In order to address those specific shortcomings, third-generation devices consisted of intracorporeal expandable titanium mesh implants for fracture reduction and maintenance of reduction. The titanium cage achieves reduction through a mechanical working system with the titanium mesh expanding into the vertebral body causing compaction of the trabecular bone. This restores vertebral body height which in turn reduces kyphotic deformity. PMMA cement is used to fill the device. Studies suggest interdigitation of the PMMA cement may be important to bone healing and structural integrity [11].

Currently, the International Myeloma Working Group (IMWG) and National Institute of Clinical Excellence (NICE) advocate the use of either balloon kyphoplasty or vertebroplasty in the management of VCFs in myeloma [12]. The majority of research involves investigating the use of expandable titanium mesh implants in single-level VCFs. We planned to review the outcome of expandable titanium mesh implants for the treatment of multi-level VCFs caused by MM. This study aimed to ascertain whether this third-generation implant is a viable alternative to older vertebral augmentation techniques and devices.

Our primary objectives were to review the complication and safety profile of the Osseofix implant and to ascertain whether this implant improves patient-reported outcomes. Secondary objectives involved investigating vertebral height restoration and deformity correction or progression following surgery.

## Methods and materials

Patients who were diagnosed with MM and VCF between 2016 and 2018 were initially included in our data collection. The IMWG and NICE indications for vertebral augmentation were applied and patients who fitted the “absolute indication” criteria for cement augmentation were treated with the Osseofix^®^ implant (Table 1) [13].

**Table 1:** Indications for surgical augmentation based on the IMWG Consensus Statement.

Primary: severe pain present (pain > 7/10 on VAS)	Secondary: severe pain absent (pain ≤ 7/10 on VAS)
Collapse of one or more vertebra (VCF)	Significant loss of height and/or structural integrity or stability
Bone destruction (osteolytic/osteopenic) with high risk of collapse of one or more vertebra	

Sixteen patients were included, 12 (75%) males and 4 females. Mean age at surgery was 62.4 years (SD = 11.6) with a median of 64 days (IQR 39–99) between imaging and operation. The median preoperative American Society of Anaesthesiologists (ASA) class was three (IQR 2–3). Twelve of our patients were diagnosed with IgG kappa myeloma, two were diagnosed with IgA lambda and two were diagnosed with IgA lambda myeloma. None of our patients received radiotherapy as there was no acute cord compression. Chemotherapy treatment consisted of Velcade, cyclophosphamide and dexamethasone (VCD) in nine patients as a single therapy, in combination with cyclophosphamide, thalidomide and dexamethasone (CTD & VCD) in five patients, melphalan in one patient and melphalan and VCD in one patient. Bracing was employed in the “conservative phase” of treatment only with no post-operative immobilisation.

Patients were then followed up for one year after their operation. Radiological follow-up included measurement of the pre- and post-operative sagittal alignment and vertebral height. The thoracic Cobb angle (angle intersected between the T4 and T12) was used as a more reliable method of assessing deformity correction instead of segmental Cobb angle due to the multi-level involvement.

**Figure 1:**
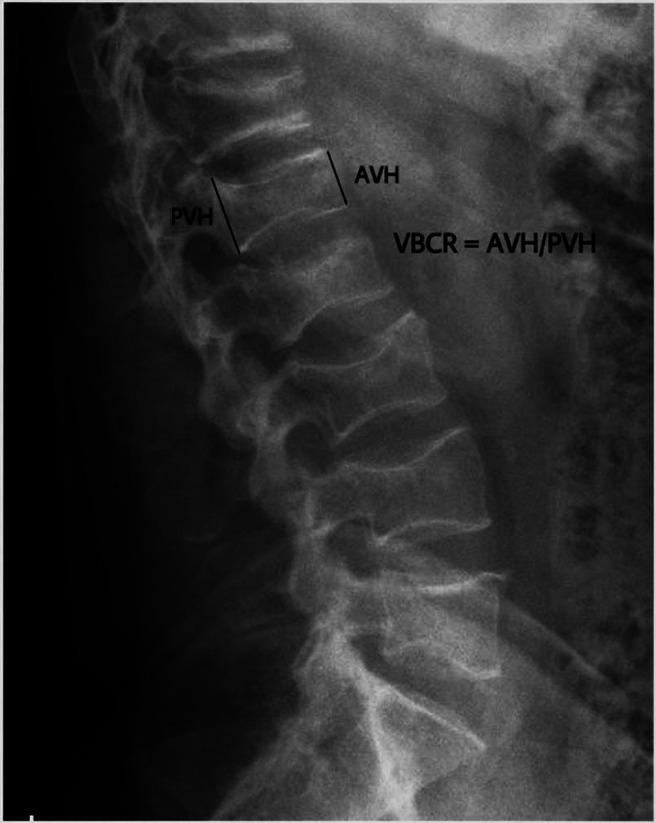
Plain radiographs were obtained before and after the surgery. We used the vertebral body compression ratio (“VBCR”), i.e. the ratio of anterior vertebral height (AVH) to posterior vertebral height (PVH) with the formula VBCR = AVH/PVH. This resulted in a percentage which was used as a parameter for vertebral body height loss.

In addition to these measurements, the vertebral compression ratio (VCR) was calculated. The percentage of the lowest height at the fractured vertebrae body using the mean upper and lower vertebral body’s lowest height of the lesion was used for the compression ratio calculation (Figure 1). As all our patients belonged to more than one level, an average VCR was taken to estimate the global correction.

Clinical follow-up consisted of pain assessment using the visual analogue scale (VAS) preoperatively and 12 months following the operation. The Oswestry disability index (ODI) is a validated questionnaire used to quantify disability for low back pain. Patients completed this questionnaire preoperatively and at the one-year outpatient follow-up.

Clinical and operative notes were reviewed for intra and post-operative complications such as cement leak, implant failure and venous thromboembolism.

### Surgical technique and details

**Figure 2:**
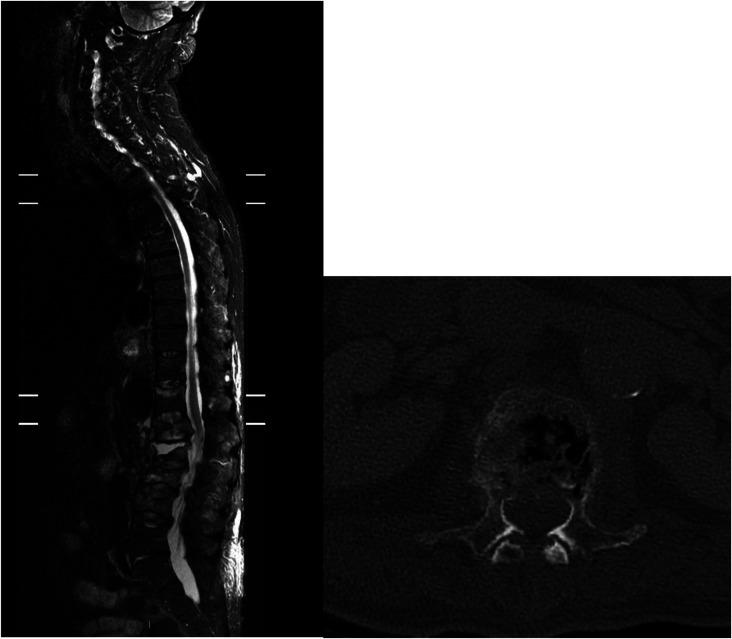
Left image – pre operative sagittal MRI STIR showing vertebral compression fractures of T12–L4 caused by myeloma infiltration. Right image – Pre-operative Axial CT scan of L2 showing a typical posterior wall defect.

**Figure 3:**
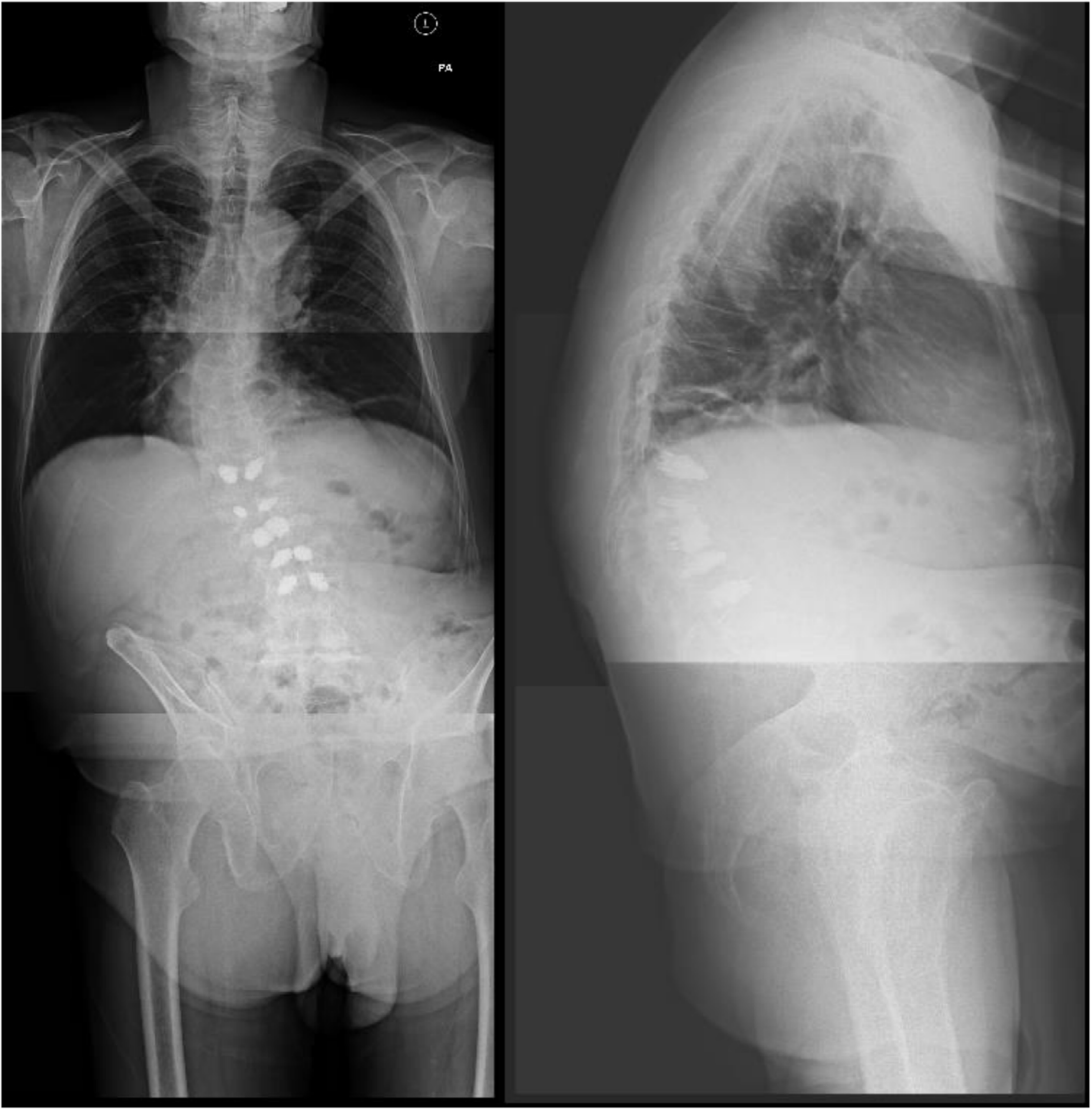
Six week postoperative PA and lateral whole spine radiographs showing multi-level instrumentation with the Osseofix™ implant.

**Figure 4:**
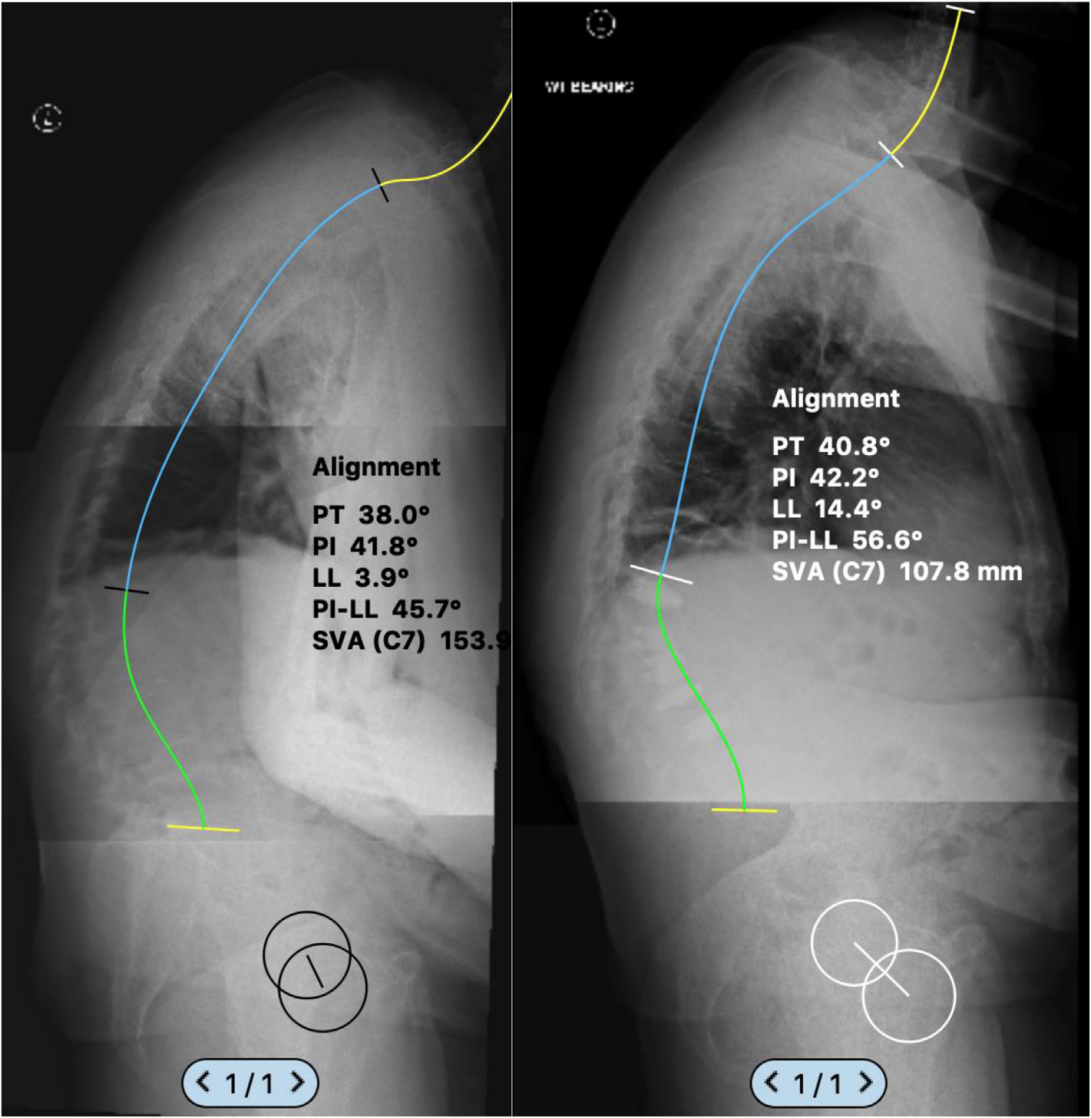
Pre and 2 years postoperative lateral whole spine radiographs showing improvement in sagittal balance after augmentation (T12-L4) with the Osseofix™ implant. using Surgimap™ software (PT = pelvic tilt, PI = pelvic incidence, LL = Lumbar lordosis, SVA = sagittal vertical axis).

The Osseofix^®^ Spinal Fracture Reduction system (Alphatec Spine Inc., Carlsbad, CA, USA) was used in all cases. Operative levels were marked using a single “C” arm and transverse stab incisions were made lateral to the pedicle. A Jamshedi needle was inserted under X-ray guidance in the AP and lateral plane into the vertebral body. A guide wire is then inserted replacing the trocar of the Jamshedi needle, and a cannulated drill (matching implant size) is threaded over the guide wire and advanced forward under X-ray control to reach the anterior third of the vertebral body in the lateral view. The drill is then removed, and the unexpanded implant is inserted in lateral view with the guide wire still in place. Once optimum position is confirmed, the guide wire is removed, and the implant is expanded. With the working cannula in place, cement is slowly injected under X-ray control. A maximum of 1 mL of cement is required to fill the expanded implant. Figures 2, 3 and 4 show a typical pre- and post-operative radiograph of a patient with multiple VCFs who underwent vertebral augmentation with the Osseofix™ implant.

Surgical details are presented in Table 2. Eighty two levels were augmented with a mean of 5 levels per patient (SD = 1.4). One hundred and 52 implants were applied with a mean of 10 implants per patient (SD = 2.9). Augmentation was performed in the thoracic spine in 5 patients (31.3%), lumbar spine in 1 (6.3%), and both the thoracic and lumbar spine in 10 (62.5%). Bipedicular augmentation was performed in 69 (84.1%) vertebral bodies. All patients were subjected to multi-level augmentation and the median length of stay postoperatively was 1.5 days (IQR 1–3).

**Table 2:** Surgical level and laterality of augmentation and cage placement in 82 vertebrae.

Patient #	Surgical level															
	T3	T4	T5	T6	T7	T8	T9	T10	T11	T12	L1	L2	L3	L4	L5	Total
1										B	B	B	B	B		5
2				B	R	B	B	B	B	B						7
3								B	B	B	B	B				5
4					B	L		B		B						4
5			B		B			B		B	B					5
6											B	B	B			3
7	L	L			L			B	B	B						6
8			B	B	B	B	B	R								6
9			B	B		B		B		B						5
10								B	B	B	B	B	B	B	B	8
11			L							B		B		B		4
12								L	B	B	B					4
13										B	B		B		B	4
14										B	B	B	B			4
15			L			L		L		L			B			5
16									L	B	B	B	B	B	B	7
Total	1	1	5	3	5	5	2	10	6	14	9	7	7	4	3	82

B = bilateral; R = right; L = left.

### Statistical analysis

The Wilcoxon signed-rank and paired *t*-tests were used to examine longitudinal changes in the primary outcome measures: kyphotic angle, VAS and ODI, depending on the distribution of data. Linear regression, using a forward stepwise approach, was utilised to model the postoperative changes in VAS and ODI and predictive baseline variables and radiological changes. Statistical significance was set at *p* < 0.05. Statistical analyses were performed in SPSS v24.0.

## Results

There were no perioperative or one-year post surgery complications necessitating re-intervention. This included no cement leakage or adjacent-level fractures and no implant failure or migration at one-year follow-up.

**Figure 5:**
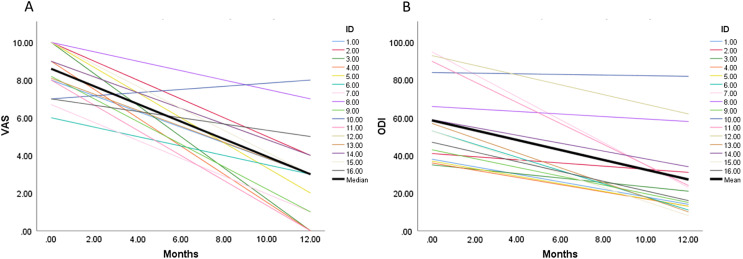
Profile plots of ODI and VAS changes 12 months postoperatively. The individual coloured lines are representative of individual patients. The bold black lines represent changes in overall size effect. The median preoperative VAS was 8.6 (IQR 7.3–10.0) improving to 3 (IQR 1.0–4.0) (*p* < 0.001, Wilcoxon signed rank test). Similarly, ODI improved from 58.6 (SD = 21.6) to 27.2 (SD = 21.7) (*p* < 0.001, paired *t*-test).

The VAS and ODI were both significantly improved 12 months post-operatively (*p* < 0.001) (Figure 5). The increased number of implants used was predictive of ODI (*B*-coefficient −4.29 [95% CI −7.29 to −1.29], *p* = 0.008) and VAS (*B*-coefficient −0.48 [95% CI −0.97 to −0.002], *p* = 0.049) improvement (Table 3).

**Table 3:** Results of linear regression modelling of VAS and ODI.

Variable	VAS		ODI	
	*B* coefficient (95% CI)	*P*	*B* coefficient (95% CI)	*P*
Age*	0.09 (−0.12 to 0.16)	0.755	0.02 (−0.93 to 1.01)	0.933
Sex (reference: female)	0.12 (−2.91 to 4.25)	0.696	0.15 (−18.35 to 31.35)	0.584
ASA*	0.01 (−2.51 to 2.61)	0.967	0.41 (−3.56 to 29.04)	0.116
Time from imaging to operation*	0.11 (−0.02 to 0.03)	0.698	−0.07 (−0.19 to 0.15)	0.814
Total levels*	−0.48 (−2.03 to 0.05)	0.061	−0.45 (−13.88 to 0.86)	0.079
Total cages*	−0.50 (−0.97 to −0.02)	0.049	−0.63 (−7.29 to −1.29)	0.008
Preoperative kyphotic angle*	0.24 (−0.04 to 0.10)	0.374	0.16 (−0.36 to 0.65)	0.551
Change in kyphotic angle*	−0.08 (−0.22 to 0.17)	0.782	−0.32 (−2.05 to 0.53)	0.225
Average VCR correction	−0.26 (−0.72 to 0.26)	0.329	−0.07 (−3.95 to 3.14)	0.809

* Entered into model as continuous variables.

As only one variable tested significant in both models, forward stepwise regression incorporating multiple variables was not feasible.

The median preoperative kyphotic angle was 39.5° (IQR 24.6–55.8) which increased to 43.9° (IQR 24.9–61.8) post-operatively; however, this did not reach statistical significance (*p* = 0.215, Wilcoxon signed-rank test). The average VCR correction was 1.5% (SD = 3.2) and there was no association between VCR and VAS or ODI. The VAS and ODI were both significantly improved 12 months post-operatively (*p* < 0.001) (Figure 5). The increased number of implants used was predictive of ODI (*B*-coefficient −4.29 [95% CI −7.29 to −1.29], *p* = 0.008) and VAS (*B*-coefficient −0.48 [95% CI −0.97 to −0.002], *p* = 0.049) improvement (Table 3).

## Discussion

Myeloma is a known cause of painful VCFs at multiple levels which can affect quality of life greatly. After conservative management has failed, options can include vertebral augmentation procedures. This study has reviewed the outcomes of a third-generation vertebral augmentation device known as Osseofix, an expandable titanium mesh implant. In our study, we have reported no complications and a statistically significant improvement in patient-reported outcomes at one year following surgery.

**Figure 6:**
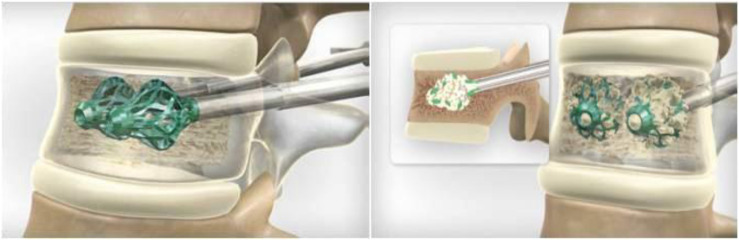
Osseofix™ implant being deployed and compacting trabecular bone and allowing improved cement interdigitation.

Both VP and BKP techniques can cause leakage of bone cement especially when the integrity of the posterior wall is lost. This can cause serious complications such as remote organ embolism, local chemical irritation or neural compression and adjacent level fractures [14–16]. From the 152 implants used, there were no clinically significant cement leakages. This resulted in a 0% leakage rate which is far superior to rates of 20–70% for vertebroplasty (3% symptomatic) and 4–13.4% (1.3% symptomatic) for kyphoplasty [17] or 6.1% for radiofrequency kyphoplasty [18]. Expandable titanium implants such as the Osseofix^®^ implant need smaller amounts of cement (0.6–1.0 mL) to augment the vertebral body. Upasani et al. have also shown that despite using less cement, expandable titanium implants are biomechanically equivalent to kyphoplasty thereby reducing the risk of cement leakage in vertebral bodies with posterior wall defects [19]. The surrounding trabecular bone is compacted outward by the device (Figure 6). This operation has two advantages: the compaction of the trabecular bone leads to a theoretical increase in the vertebral body height, thus reducing kyphotic deformity, and the interdigitation of trabecular bone into the mesh stabilises the system itself. After this process, cement is injected inside the expanded device; significantly less PMMA is required in comparison to standard kyphoplasty. Moreover, the PMMA creates an interdigitation between the cells of the titanium mesh and the surrounding bone, further stabilising the system and preventing cement leakage [20]. This implant has also been advocated in the use of symptomatic osteoporotic stable burst fractures. Ender et al. have shown favourable clinical results with reduced rates in terms of cement leakage and secondary vertebral subsidence [20]. With the number of osteoporotic VCFs expected to double by 2050, it is important to acknowledge other methods of vertebral stabilisation [21].

A significant improvement in patient-reported outcomes was noted. Patients experienced significantly less pain twelve months postoperatively as shown by a 65% improvement in VAS score. Similarly, ODI improved from 58.6 (SD = 21.6) to 27.2 (SD = 21.7). Our study, therefore, found comparable results to kyphoplasty and vertebroplasty after 12 months in the functional outcome (ODI) and pain relief (VAS). Regression analysis of the data found a statistically significant relationship between the number of implants used and improved ODI and VAS. We hypothesise that patients who have multi-level VCFs have more pain and worse function. As these levels are augmented and stabilised, they offer the patient greater relief of symptoms and improved function. Based on these results, we would advise early augmentation surgery for the treatment of multilevel VCFs.

We found that the median kyphotic angle progressed from 39.5° to 43.9° postoperatively, but the average single level VCR improved by 1.5%. Ender et al. outlined improvement in the local kyphotic angle following implantation of expandable titanium mesh implants for single-level osteoporotic fractures [20]. Our study involved patients who required a mean of 5 levels of augmentation and therefore represented a more severe disease process and spinal deformity. Despite the perceived worsening of kyphosis in our study, this was not statistically significant. There was also no relation between change in deformity and outcome scores showing that this device offers pain relief and improved function but not deformity correction.

Limitations of this study include a relatively small sample size of patients. However, the total number of implants used in this study is the highest reported in the current literature. Our follow-up finished at one year and we acknowledge the long-term outcome of surgery was not included in this study. A follow-up to this study would be to perform a randomised controlled trial directly comparing vertebroplasty and kyphoplasty to the Osseofix implant.

## Conclusion

Our study represents the largest published series of titanium mesh implants used for the treatment of VCFs in myeloma. We have shown good outcomes comparable with older more established augmentation techniques with the main advantage being a greatly reduced complication profile. From the 82 implants used, there were no recorded complications such as cement leakage or adjacent-level fractures. From our experience, the more symptomatic patients suffer with multiple VCFs at various levels. This is the first study to investigate the clinical and radiological outcomes of stabilisation on multiple vertebral fractures in MM and shows an improved outcome with more levels that are augmented.

We believe that expandable titanium mesh implants are a safe and clinically effective method of relieving pain, improving function, and stabilising VCFs in myeloma patients. Such implants should be advocated by national and international organisations as another treatment strategy for these patients.

## Conflicts of interest

The authors declare that they have no conflicts of interest in relation to this article.

## References

[1] Jemal A, Siegel R, Xu J, Ward E (2010) Cancer statistics, 2010. CA Cancer J Clin 60, 277–300. 20610543 10.3322/caac.20073

[2] Tosi P (2013) Diagnosis and treatment of bone disease in multiple myeloma: Spotlight on spinal involvement. Science 2013, 1–12. 10.1155/2013/104546 PMC3870870 24381787

[3] Kulkarni AG, Patel A (2019) Denosumab: A potential new treatment option for recurrent Aneurysmal Bone Cyst of the spine. SICOT-J 5, 10. 30931897 10.1051/sicotj/2019007 PMC6442452

[4] Hoes JN, Jacobs JWG, Hulsmans HMJ, et al. (2010) High incidence rate of vertebral fractures during chronic prednisone treatment, in spite of bisphosphonate or alfacalcidol use. Extension of the alendronate or alfacalcidol in glucocorticoid-induced osteoporosis-trial. Clin Exp Rheumatol 28, 354–359. 20406615

[5] Malhotra K, Butler JS, Yu HM, et al. (2016) Spinal disease in myeloma: Cohort analysis at a specialist spinal surgery centre indicates benefit of early surgical augmentation or bracing. BMC Cancer 16, 444. 27401073 10.1186/s12885-016-2495-7 PMC4939590

[6] Vanni D, Galzio R, Kazakova A, et al. (2016) Third-generation percutaneous vertebral augmentation systems. J Spine Surg 2, 13–20. 27683690 10.21037/jss.2016.02.01 PMC5039833

[7] Molloy S, Lai M, Pratt G, et al. (2015) Optimizing the management of patients with spinal myeloma disease. Brit J Haematol 171, 332–343. 26184699 10.1111/bjh.13577

[8] Saracen A, Kotwica Z (2016) Complications of percutaneous vertebroplasty: An analysis of 1100 procedures performed in 616 patients. Medicine 95, e3850. 27310966 10.1097/MD.0000000000003850 PMC4998452

[9] Luo J, Adams MA, Dolan P (2010) Vertebroplasty and kyphoplasty can restore normal spine mechanics following osteoporotic vertebral fracture. J Osteoporos 2010, 729257. 20981329 10.4061/2010/729257 PMC2957176

[10] Krüger A, Oberkircher L, Figiel J, et al. (2013) Height restoration of osteoporotic vertebral compression fractures using different intravertebral reduction devices: A cadaveric study. Spine J Official J North Am Spine Soc 15, 1092–1098. 10.1016/j.spinee.2013.06.094 24200410

[11] Schützenberger S, Schwarz SM, Greiner L, et al. (2018) Is vertebral body stenting in combination with CaP cement superior to kyphoplasty? European Spine J Official Publ European Spine Soc European Spinal Deformity Soc European Sect Cerv Spine Res Soc 27, 2602–2608. 10.1007/s00586-018-5717-7 30099668

[12] National Institute for Health and Care Excellence (2013) Percutaneous vertebroplasty Interventional procedures guidance [Internet]. London: NICE. https://www.nice.org.uk/guidance/IPG12.

[13] Hussein MA, Vrionis FD, Allison R, et al. (2008) The role of vertebral augmentation in multiple myeloma: International Myeloma Working Group Consensus Statement. Leukemia 22, 1479–1484. 18509352 10.1038/leu.2008.127

[14] Walter J, Haciyakupoglu E, Waschke A, et al. (2011) Cement leakage as a possible complication of balloon kyphoplasty – Is there a difference between osteoporotic compression fractures (AO type A1) and incomplete burst fractures (AO type A3.1)? Acta Neurochir 154, 313–319. 22146845 10.1007/s00701-011-1239-3

[15] Nieuwenhuijse MJ, Erkel ARV, Dijkstra PDS (2011) Cement leakage in percutaneous vertebroplasty for osteoporotic vertebral compression fractures: Identification of risk factors. Spine J Official J North Am Spine Soc 11, 839–848. 10.1016/j.spinee.2011.07.027 21889417

[16] Omidi-Kashani F, Samini F, Hasankhani EG, et al. (2013) Does percutaneous kyphoplasty have better functional outcome than vertebroplasty in single level osteoporotic compression fractures? A comparative prospective study. J Osteoporos 2013, 690329. 23970997 10.1155/2013/690329 PMC3732604

[17] Röllinghoff M, Zarghooni K, Dargel J, et al. (2010) The present role of vertebroplasty and kyphoplasty in the treatment of fresh vertebral compression fractures. Minerva Chir 65, 429–437. 20802431

[18] Feng L, Shen J-M, Feng C, et al. (2017) Comparison of radiofrequency kyphoplasty (RFK) and balloon kyphoplasty (BKP) in the treatment of vertebral compression fractures: A meta-analysis. Medicine 96, e7150. 28640091 10.1097/MD.0000000000007150 PMC5484199

[19] Upasani VV, Robertson C, Lee D, et al. (2010) Biomechanical comparison of kyphoplasty versus a titanium mesh implant with cement for stabilization of vertebral compression fractures. Spine 35, 1783–1788. 20098352 10.1097/BRS.0b013e3181b7cc5d

[20] Ender SA, Eschler A, Ender M, et al. (2015) Fracture care using percutaneously applied titanium mesh cages (OsseoFix^®^) for unstable osteoporotic thoracolumbar burst fractures is able to reduce cement-associated complications – results after 12 months. J Orthop Surg Res 10, 175. 26568074 10.1186/s13018-015-0322-5 PMC4644291

[21] Stein KV, Dorner T, Lawrence K, et al. (2009) Ökonomische Konzepte zur Erfassung der Krankheitskosten von Osteoporose: Österreich im internationalen Vergleich. Wien Med Wochenschr 159, 253–261. 19484209 10.1007/s10354-009-0674-8

